# Spaces for skateboarding in the city–new spatial concepts beyond skateparks

**DOI:** 10.3389/fspor.2024.1457427

**Published:** 2025-02-14

**Authors:** Veith Kilberth

**Affiliations:** Institute of Sports Science at Europa-University Flensburg, Cologne, Germany

**Keywords:** skateboarding, skatepark, skate park, urban planning, action sports, public spaces, landscape architecture, creative city

## Abstract

Since the 1990s, skateboarding has emerged as a significant urban practice, often resulting in spatial conflicts. The predominant response from municipal authorities has been to confine skateboarding to purpose-built skateparks, overlooking more integrated and inclusive spatial solutions. This study critically examines this approach and explores alternative skateboarding spaces within the framework of urban sociological discourse on the creative city phenomenon and the evolving collaboration between skateboarding communities and city authorities. Employing a praxeological approach, the analysis integrates cultural theory, discourse analysis, and fieldwork. Building on existing literature, the study is complemented by case study analyses of skateboarding spaces worldwide. To provide a structured understanding, a spatial typology is developed, encompassing purpose-built skateparks, self-constructed *DIY* projects, *shared spots*, and *legalized street spots*. These spaces are conceptualized along the axes of exclusion vs. inclusion and subcultural vs. sportification. Key opportunities and essential conditions for the implementation of innovative spatial concepts in urban environments are identified, with particular emphasis on the pivotal role of collaboration between skateboarding communities and municipal authorities. By presenting a theoretical framework for diversifying skateboarding spaces, the findings contribute to the urban planning discourse and promote participatory urban development and design.

## 1 Introduction

Ever since street skateboarding became the dominant discipline at the beginning of the 1990s and the skateboarding scene's field of activity began to focus on urban architecture, spatial conflicts have increasingly arisen in cities. Conflicts such as noise pollution, damage to property, traffic hazards, and trespassing have often arisen and continue to persist. As a result, more and more found skate spots^1^ in public spaces have since been thwarted by structural measures and bans. The exclusion of skateboarding from public spaces by means of skateboard bans and “defensive architecture” (1), among other aspects, is still problematic today and has been the subject of critical debate for over two decades (1–4). In response to the increased number of participants and the regulatory displacement of skateboarding from urban areas, purpose-built spaces in the form of skateparks are being built worldwide (5–8).

**Table 1:** *Skateboarding space matrix*.

	Found space	Purpose-built space	Self-built space	Approved, supported, or built by city
Found space	e. g. *Street spot*	*Shared spot*	*DIY illegal*	*Street spot legalized*
Purpose-built space	*Shared spot*	Skatepark	*DIY hybrid*	skatepark and *Shared spot*
Self-built space	*DIY illegal*	*DIY hybrid*	*DIY*	*DIY legalized* and *DIY hybrid*

Source: Own presentation.

It is important to note that the skatepark development should not be regarded as a singularly successful evolution. The process of sportification and acculturation of street skateboarding began with the urbanization of urban movement practices in skateparks as functional facilities. In the scientific community, skatepark development gives rise to critical debates. There is talk of “panoptical architecture” (9), the “domestication” (10) of street skateboarding or even “fenced-in pens” (11). The creation of skateboarding facilities and the reference of law enforcement officers to such facilities goes hand in hand with a criminalization of the practice in urban spaces. The practical immanence of this causal relationship is easy to understand and has been proven several times by scientific studies (3, 9, 12).

This study aims to contribute to the discourse on the relationship between the skateboarding community and the city. The focus is on the ongoing process of negotiating where in the city skateboarding has its legitimate spaces. After all, how and where spaces for skateboarding are created by the city does not necessarily have to coincide with the ideas of the skateboarding scene. From a critical perspective, the urban policy approach in the form of skateparks—representing society—could also be interpreted as a lack of acceptance of skateboarding. Simultaneously with the development of skateparks, it can be observed that there is also an increased emergence in quality and quantity of new spatial concepts for skateboarding like *DIY-hybrid*, *shared spot*, s*treet spot legalized* and more. These approaches are different as they have fundamentally more including characteristics. This raises the question what are these characteristics exactly and whether the relationship between the city and skateboarding may has changed?.

### 1.1 Objective

The objective of this study is to expand the mostly one-dimensional approach of city politics to build skateparks as spaces for skateboarding and to conceptualize new options for action that highlight both socio-political opportunities and difficulties.

From the segregation of skateboarding in purpose-built spaces, the article aims to explore opportunities of spatial concepts that “reintegrate” skateboarding back into the public realm as structurally more integrated and inclusive spaces in the city.

Using a selection of these innovative spaces as *models of good practice*, the study intends to analyse, from a city political and urban sociological point of view, what are the enabling and constraining factors.

Based on these practical examples, it is then to define characteristics of the new spatial concepts and to derive a theoretical framework on a structural level, that lead to a positioning model of spaces for skateboarding as a comprehensive overview.

This study contributes to the academic discourse by offering a broader perspective for understanding and analyzing the emergence of new spaces for skateboarding. It is considered fundamental research that addresses a gap in skateboard studies and lays the foundation for future investigations. Building on the concepts discussed under the keywords “skateboard urbanism” (13), “city of play” (14, 15) and “city play” (16), this study aims to provide a scientifically grounded, practical guide that expands the range of options for citizen initiatives by active users advocating for, and municipalities deciding on, appropriate skateboarding spaces within their cities.

The objective of the study leads to a twofold research question: (1) How can new concepts of spaces for skateboarding be scientifically defined and conceptualized? (2) What are the enabling and limiting factors?.

## 2 Method

Addressing the complex research question requires foundational groundwork to inform the theory-driven investigation. For this theory-based study, a praxeological approach was employed (17). This methode integrates cultural studies theories, incorporates elements of discourse analysis, and is informed by both direct and indirect practical observations from fieldwork, as well as *models of good practice* case studies (18). Practical observations and case study examples serve as essential prerequisites for understanding and interpreting the contexts in which the praxeological method is applied.

### 2.1 Theories from cultural studies and elements of discourse analysis

A significant portion of this research involves theoretical analysis and interpretation of cultural studies literature, with a particular focus on skateboarding studies and the subject of skateboarding spaces. It builds upon English-language research while also incorporating contributions from German scholars in the field of skateboarding studies (e.g., Peters, Schäfer, Schweer, Schwier), thereby enriching the international discourse.

A similar approach was utilized to adopt a broader perspective on the political and urban sociological development of cities. The theoretical framework is based on numerous sources, with particular reference to the highly regarded work of German sociologists Martina Löw and Andreas Reckwitz.

The scientific literature is supplemented by relevant skateboard-specific media and other non-academic sources on urban development, where necessary.

### 2.2 Practical observations, fieldwork and case study analysis

The relevant fieldwork observations were conducted alongside my work as a skatepark planner over the course of eight years. I am the co-owner of a landscape architecture firm that specializes in designing urban spaces, with a primary focus on skateparks. Through my direct collaboration with cities, municipalities, and skatepark advocates in the planning of public skateparks, I have drawn from numerous encounters. To quantify this experience, my professional work has involved contact with over 100 skatepark projects, approximately 2,000 participants, and communication with more than 300 city officials.

Although the fieldwork was not explicitly aimed at collecting empirical data, relevant categories of observation such as the wants and needs of user groups, the objectives, opportunities, and limitations of city officials, as well as broader trends and new developments were reconstructed as specific background knowledge for this study.

The selection of *models of good practice* as case study samples is based on Peters' “Typology of Skateboarding Practices” (9) and was expanded to include non-purpose-built spatial types, such as *shared spots*. The selection process was carried out using a deductive scientific approach to identify predefined spatial concepts with existing examples that meet specific structural criteria. Additional criteria were established to encompass a broad range of factors, including the presence and perceived significance of these spaces within the skateboarding community, as well as the availability of publicly accessible information relevant to the analysis.

### 2.3 The interpretative approach for data analysis and theory development

For theory development and data analysis, a hermeneutic-interpretative research paradigm (19) is applied. Hermeneutics, traditionally rooted in philosophy, encompasses various currents (20). It is more accurately understood as a structural tool rather than a research method. As a structural technique, hermeneutics offers a range of theories, rules, approaches, and procedures for the analysis of texts and other sources, with the aim of facilitating deeper understanding, particularly in social science contexts (18). The application of a hermeneutic approach requires comprehensive field knowledge as a fundamental prerequisite for understanding and interpreting contexts within an interpretative framework (ibid.). The following section exemplifies three central rules of hermeneutics, as outlined in Danner's (21) concept:

First Rule: Meaning: Understanding is the central focus of hermeneutics. Its primary task is to comprehend a statement in terms of its meaning and significance. While the natural sciences paradigmatically emphasize deductive explanations of causal relationships, hermeneutics is concerned with inductive understanding (22).

Second Rule: Objective Spirit and Objectivity: For an impartial understanding, the concept of Objective Spirit serves as a key prerequisite. It is essential to emphasize that meaning must be derived from the subject being interpreted, rather than imposed upon it (18). The interpreter must remain conscious of their own preconceptions and biases, and critically assess their interpretation with objectivity. In addressing the issue of the universality and neutrality of understanding, it is important to clarify that an objective interpretation of meaning should not be conflated with absolute truth. “Truth is defined as the alignment of knowledge with its object” (ibid.).

Third Rule: Hermeneutic Circle: The hermeneutic circle is a core principle in hermeneutics, explaining the process of understanding a text or phenomenon by constantly shifting between its parts and the whole. It posits that to fully grasp the meaning of a specific element, one must first understand the broader context, and conversely, to comprehend the whole, attention must be given to its individual components. In this interpretive process, understanding is not linear but circular: the significance of each element (such as words, sentences, or concepts) is revealed through its relationship to the entire context, while comprehension of the whole is continually refined by the analysis of its constituent parts. This reciprocal interaction fosters a deeper and evolving insight into both the individual and the collective dimensions of the subject being examined.

In conclusion, hermeneutics embodies a foundational approach and incorporates a variety of interpretive techniques that promote a clearer and more objective understanding of new data and information. The explicit use of different hermeneutic strategies, when applied to the sources under examination, is always guided by their appropriateness to the context.

### 2.4 Potential researcher bias in the study

It must be acknowledged that occupying multiple roles—both as an active participant and a researcher in the field—can provide significant advantages in terms of data access and collection. However, this dual involvement may introduce challenges related to subjectivity (23). To mitigate this inherent issue, I follow Reichertz (24), who emphasizes transparency as the key principle of scientific research: “Research must remain transparent; this is the fundamental criterion for the quality of research” see also (25).

To ensure transparency and allow the reader to better comprehend, evaluate, and critically engage with my arguments and conclusions, I will concentrate on three main aspects: (1) biographical context, (2) personal motivations, and (3) potential challenges encountered by researchers.

(1) Biographical information^2^ and (2) motivation: The fieldwork effectively began when I started skateboarding in 1987 and continues to this day as an active participant. As a former professional skateboarder with over three decades of involvement in the skateboarding community, I have travelled globally, skating in a variety of spaces, both those intentionally built for skateboarding and many that were not. I have personally experienced most of the *models of good practice* presented in this study. Transitioning from a professional skateboarder to the business side of the industry, I see myself as serving a “bridge function” between the skateboarding space advocates and city officials (as well as between the skateboarding community and the academic world). In terms of my academic background, I hold a diploma in sports science and wrote my dissertation on skateparks within the field of sports sociology. I have a strong understanding of the user's perspective, as well as unique insights into the perspectives of municipal politicians and employees who make decisions about skateboarding spaces. My motivation for this scientific endeavour is to leverage my unique position, expand the range of potential skateboarding spaces, and thereby improve the situation for both the skateboarding community and cities.

(3) Potential challenges faced by researchers: Among the various types of potential errors, I will highlight four significant “deceptions as a researcher” (8) that warrant attention: (A) bias resulting from the researcher's idealistic assumptions; (B) partiality influenced by personal preferences regarding skateboarding space concepts; (C) conflicts of interest arising from professional or economic considerations; and (D) the risk of confirmation bias. The first step in addressing these challenges is a conscious, self-reflective approach. For a more in-depth reflection on how subjective experiences can be made fruitful as a source for academic work, I refer to my monograph (8).

### 2.5 Scope of research

There are many studies on spaces for skateboarding (see below). To address aspects that have not yet been covered, I must limit my research scope geographically (industrialized democratic vs. non-democratic countries), politically (public vs. private skateparks), by type of sport (skateboarding vs. action sports), and structurally (spatial material vs. programming of skateparks).

### 2.6 Structure of the article

The article commences with an examination of the complex relationship between skateboarding and the urban landscape, beginning with an in-depth analysis of the skatepark dilemma. This is followed by a discussion that situates the concept of the *creative city* within the broader framework of the evolution of the skateboarding community. New and innovative spatial concepts are defined, and exemplary *models of good practice* for the creative and functional integration of skateboarding spaces into the urban fabric are presented, with a focus on their respective advantages and challenges. These spatial concepts are subsequently positioned within a comprehensive model that delineates the spectrum between exclusion and inclusion. The article concludes by critically assessing the possibilities and challenges inherent in these spaces, followed by a summary of the key findings and an outline of potential future research avenues.

## 3 The ambivalent relationship between skateboarding and the city

### 3.1 The skatepark dilemma

The skatepark dilemma stems from the positive aspects that skateparks provide for the city and skatepark users by simultaneously structurally diminishing the situation for other user groups.

In the contemporary era, the construction of public skateparks is experiencing a period of significant growth. From major metropolises to the smallest villages in rural regions, the demand for urban recreational spaces is growing worldwide. There are many factors behind the success of skateparks, foremost among them being a predominantly participatory design process involving local user groups, which enhances both the design quality and the overall utilization. Another key aspect is that the benefits of skateparks usually go beyond the needs of the skateboard scene and state-of-the-art skateparks often serve a wide range of social aspects. Skateparks are often regarded as safe spaces for skateboarding^3^ and offer a range of positive aspects. They promote health benefits (26), provide dedicated spaces for skateboarding, enhance accessibility, often serve as low-threshold opportunities, particularly for beginners, and can function as extracurricular learning environments (8, 27, 28). In addition, skateparks often offer an infrastructure for other youth-cultural movement practices such as BMX, inline skating, stunt scooters and, for some years now, *WCMX* (wheelchair *MotoX* or skating) (29), which qualifies them as multifunctional spaces. As a result of social negotiation processes in the pursuit of funding for public skateparks, the spatial structure of many skatepark designs ideally has the tendency to show the following three characteristics, which are intended to maximize the social benefits for the general public: “(1) multifunctionality”—skateparks for a broad user group; “(2) intergenerationality”—skateparks as safe places for young and old and “(3) interperformativity”—skateparks for every skill level (8). Consequently, on an international level, “over the last two decades, a veritable skatepark renaissance has been underway.” (30). Obviously, the spatial concept of the skatepark as a functional facility seems to be the favored solution for dealing with the above-mentioned skateboard space conflict in cities. It is important to mention that in these primarily self-regulated social spaces, there are power dynamics at play that can result in unequal access to and use of the space, which may be particularly problematic for marginalized user groups (31–33).

From a different perspective, the distribution of skateparks can also be seen critical. There is no doubt that the creation of skateboarding opportunities using modern *in situ* concrete skateparks is perceived positively by many stakeholders (e.g., *skatepark advocates*) and that reference can be made to the aforementioned social aspects. The protagonists of skatepark projects nolens volens become part of a development that is also described socially critically as a civilization process (27, 34, 35), in which they themselves have in part unwittingly participated (36). As a result, “skateboarding […] has become a piece of urban life that tries to assert itself in neoliberal displacement scenarios” (37) and finds itself in a self-relationship “between urban rebellion and neoliberal self-design” (36). If we characterize the regulation using skateparks as a taming of the free “body-space game” (38) of street skateboarding in the city, then skateparks can create access for other social groups, but this can also lead to a dilemma: The more the social benefits increase through the creation of skateparks, the more the opportunities for free use of the city's space decrease. The openness of interpretation of the practice and the spatial freedom of skateboarding are to a certain extent forfeited in favor of the social benefits of purpose-built spaces. In addition, there is a growing phenomenon of a new spatial conflict that occurs in skateparks as multifunctional spaces. Namely, when the skatepark becomes a place “for the whole family” (8); see also (27, 39) and new user groups, such as young stunt scooter users and their parents, demand a socially legitimized claim to the space (40–43).

The solution to this *one users gain is another users loss-*dilemma can only lie in a diversity of spaces for skateboarding—this much can be said in advance—by emphasizing the creation of different types of space, but this presupposes a socio-cultural understanding of the need for street skateboarding in public spaces and participation in urban life (12, 44, 45), which can by no means be taken for granted.

With the intention to discuss alternative spatial concepts in the city to complement skateparks, the relationship between skateboarding and the city must first be outlined.

### 3.2 The relationship between skateboarding and the city

City politics and urban sociology create the broader context in which new spaces for skateboarding are either facilitated and supported or hindered from coming into existence.

The relationship between skateboarding and the city is sometimes very complex and takes many different forms. It is based on a variety of prerequisites, relates to different success factors and cannot be easily conceptualized in an all-encompassing way. Without the following explanations being able to claim undifferentiated validity for all cities and municipalities, the central development trends are now roughly outlined and worked out using a few indicative and directional examples of skateboarding space concepts as well as a possible genesis of municipal skateboarding space offers and a skateboarding space concept positioning model.

On the subject of the relationship between skateboarding and the city, two phenomena can be observed that have developed independently of each other and are related to each other here. Of the city's diverse economic, social and political discourses, the highly simplified, generalized orientation towards the model of the *creative city^4^* appears to be of particular importance. The other development is a change in the public perception of skateboarding in many places and an increased willingness of the skateboard scene to engage in political processes and to collaborate with city administrations (see below).

### 3.3 The concept of the *creative city*

Cities are undergoing change in the context of profound social upheaval. New answers are increasingly being found to the socio-political question of how cities can meet the desires and needs of their citizens during social change, in order to become more livable and attractive, while coping with the pressures of modernization and competition. According to Schäfer, in the context of the skateboarding space issue, “the transition from the so-called neo-realism of the 1990s—from the policy of zero tolerance—to the guiding principles of the *creative city* after the turn of the millennium is particularly relevant […]” (39). Nowadays, cities are increasingly striving for creativity and elements that revitalize the city, make it unique, and enhance the overall quality of life. Various sociological studies on the contemporary diagnosis of the city come to the unanimous conclusion that many cities today rely on creativity and distinctiveness as a competitive strategy, as pointed out, for example, by “Cities and the Creative Class” (46), the “intrinsic logics of cities” (47) and “urban spaces to be culturalized and singularized” (48). This applies even more to large cities and metropolises that are affected by globalization (cf. ibid.). The striving for distinctiveness relates above all to the differentiation from other cities (47) and takes place in an external and internal relationship. In the external relationship, the *creative city* plays an important role as a potential competitive advantage due to the increasing competition between cities (48). In this competition, there is a tendency to position the city as a brand (49). As a city, increasing marketing mechanisms is not only aimed at strategically appealing to tourists and possibly strengthening the patriotic identity of the inhabitants of their city, but also at attracting potential new citizens, preferably from the knowledge and creative industries (50), which ultimately pursues economic objectives, because “Sooner or later, enticing job opportunities for highly qualified people eventually cluster in attractive cities” (48). As a result, city marketing is becoming increasingly important, especially for large cities. The phenomenon of a “local government” (ibid.) is emerging in many of these large cities that are in global competition. In other words, a policy of the city that does not have to correlate with its respective nation state and which is referred to as “glocalization” (51). Put simply: In a climate of urban policy that leans toward neoliberalism, the city takes a step back as a provider of ideas and instead seeks to channel the momentum of the creative initiatives from groups, organizations—both non-profit and, above all, for-profit companies—making it productively usable for urban planning. This political concept, which actively involves companies and citizens as co-producers in urban development, is referred to as “urban governance” (52) or liberal “governmentality” (48). Internally, one of the aims is to create a new socio-political climate within the city, from hard to soft politics. The political approach from authoritarian politics, such as a master plan that is executed *from above*, to alternative urban planning and civic participation, which is discussed under the heading of planning “*from below”* (48). This is sometimes about promoting the creative potential of city dwellers through their own development opportunities, revitalizing the city and thus creating a *people climate* in the city that is attractive to young, creative and innovative citizens (50). This is because “the attractiveness of a city [is] becoming an increasingly important location factor for young people” (53).

### 3.4 The evolution of skateboarding and the scene's willingness to collaborate

Processes of change can also be observed in skateboarding. Since the emergence of street skateboarding in the early 1990s, the phase that can be described as the “puberty of the [skateboard] scene” (54), the practice has evolved from a sometimes self-centered attitude of rebellious young people to a certain maturity (55). Today, the skateboard scene is increasingly involved in political participation in urban planning processes. In many instances of skatepark planning participation, I have observed that some of the initiators of skatepark projects often belong to the “organizational elite” (56) of the scene, many of whom are over 30 years old.^5^ Today, decades later, the generation of street skateboarders from the 1990s is correspondingly older and, in Peters' words, “part of those lifestyle skateboarders who have long since pursued socially recognized professions as teachers, architects, artists, doctors, or photographers and thus have the necessary skills to actively intervene in political decisions” (9). More and more skateboarders, especially the slightly older ones, are exercising their grassroots democratic right to have a say as citizens and are campaigning for public spaces for skateboarding—mostly skatepark projects. Overall, they are increasingly willing to collaborate with city and municipal administrations. “Increasingly, skateboarding communities are starting to work towards finding ways of including skateboarding in urban space” (57) writes the German skateboard magazine *Solo Skateboard* in a special issue on the topic of “Skate Urbanism”. Such forms of cooperation may also have contributed to the shift in public perception of politics and city administrations toward skateboarding, which, in turn, is likely to open new opportunities for collaboration.

### 3.5 The creativity of skateboarding and the *creative city*

If we now relate these two development trends, the model of the *creative city* in many large cities and the skateboard scene's willingness to collaborate, to each other, then a variety of starting points arise in the intersection of mutual needs and objectives. Various studies suggest that skateboarders are a “productive force of the urban” (6), have long been part of the creative economy (58–60) and can have a positive influence on the appearance of a city (3, 9, 39). Places where skateboarding takes place can have a vitalizing effect (38) and, as “shock troops of gentrification” (58), contribute to the upgrading of residential districts (6, 36, 61). In a sense, they are part of the city and make up the city (59, 62).

From a socio-theoretical perspective, some of the central characteristics of skateboarding can be linked to the catalog of requirements of the “late-modern working subject” identified by Reckwitz. These include “experimentalism and a playful attitude, creativity and an orientation towards the moment instead of long-term planning, constant willingness to change oneself” (63). Skateboarding may be regarded as a symbol of the non-conformism that is required of late modern subjects (48). Glenney and O'Connor (64) observe in this regard that the public perception of skateboarding has changed today in that “the subcultural creativity of skateboarding, that was once socially peripheral and even subversive, is now recognized as possessing the hallmarks of self-starting creativity necessary to weather the capricious precarity of the globalized world […]”. Cities that are fundamentally interested in promoting values such as creativity, experimentation, self-starting initiative and the like could set an example by tolerating urban practices in public spaces (especially at the limits of what is legal). After all, whether citizens are allowed to (co-)create their city in an artistic and creative way (e.g., *urban knitting*, *urban gardening*, legal graffiti walls or skateboarding) says a great deal about a city. The act of prohibiting and preventing, as opposed to tolerating and explicitly allowing, has the potential to facilitate or impede urban creative development. This, in turn, reflects certain attitudes and values of the city. A certain degree of tolerance, courage and creativity could be reflected in the use of existing found skateboarding spaces (passively) and in proactively supporting the creation of new spatial concepts, in line with the guiding principle of the c*reative city* (9). It can be argued that skateboarding could serve as a symbolic catalyst, acting as an “agent of culturalization” (ibid.), thereby contributing to the upgrading of urban space (39).

### 3.6 Skateboarding in city marketing and as an attraction for tourism

The added value of skateboarding for the city (brand) is not only seen in terms of a modern, contemporary exercise program for young people that combines sporting and social aspects to a particular degree, but goes far beyond this: from an urban policy perspective, skateboarding can have the potential to contribute to the positioning of the city brand (9, 39). Cities can utilize the positive effects of skateboarding in the realms of leisure and sport for city marketing and use it as a location factor (65). Unlike standardized sports facilities for traditional sports, individually designed skateparks and other spaces for skateboarding can develop a special attraction far beyond the geographical boundaries of cities and municipalities (27, 35). They have the potential to become places of pilgrimage for skateboarders (66).^6^ Just as modern sports architecture can contribute to the image of a city (67), this principle can also be applied to particularly striking skateparks, *street spots*, *shared spaces*, and *DIY* parks, which can serve as centers of attraction for tourism and become integral components of a *creative city*. The image factor of skateboarding can attract creative people as human capital, which can have a positive impact on the creative industries, tourism and the image of the city. Skateboarding thus becomes a soft location factor for a city that sees itself—to put it bluntly—as cosmopolitan, youthful, hip and tolerant.

### 3.7 Criticism of the *creative city* and the *creative class*

Anyone who emphasizes the advantages and possibilities of the concepts of the *creative city* and *creative class* or creative industries must also point out the general criticism that these approaches provoke. It is quite understandable that the neoliberal climate of the *creative city* and the focus on the *creative class*—i.e., the protection of a specific economic sector—gives rise to criticism regarding inequality and grievances. Essentially, the criticism relates to the fact that culture is reduced to what is economically exploitable and creativity is associated with the potential for commercialization. In accordance with the urban policy orientation, the *creative city* and *creative class* favour certain people, groups and organizations that are better off in this sense, which makes such concepts socially selective (68) and the “creative city as the antithesis of urban creativity” (ibid.). According to Mould, it contributes little to the creativity and diversity of urban design, contrary to what the label suggests. If urban policy is commercially oriented and socially selective, if the economic balance of power between the advantage of financially strong companies and the interests of city residents is not meaningfully regulated and urban “living space becomes private property” (69), the demand for a “[…] right to the city” (70) is more relevant than ever and leads to the ongoing discourse on the legitimate question: “Who owns the city?”.

## 4 Case study of examples of innovative and creative skateboarding space approaches

In the context of the *creative city* paradigm and the assumption that cities aim to promote creativity and uniqueness, these characteristics can also be applied to skateboarding spaces. Emerging spatial concepts can fundamentally deviate from the conventional design of traditional skatepark facilities. These spaces tend to be more creatively and diversely designed, vary in size and dimension, are located in unconventional areas, often repurpose existing spaces, are sometimes developed through collaborative co-creation processes, and aim to be more integrative and seamlessly embedded within the urban fabric.

The following exploration of exemplary models, presented as cursory case studies, aims to examine the phenomenon of new spatial concepts from a broader perspective, maintaining some distance to highlight a wide spectrum of occurrences of these innovative spaces. This praxeological study is regarded as a preliminary step for future empirical research that can conduct a more detailed analysis of individual examples.

### 4.1 Definition of skateboarding spaces

Among the various definitions, typologies, and categorizations of skateboarding spaces (2, 3, 5, 8, 39, 64), the criteria for analysis used here builds on Peters' “Typology of Skateboarding Practices” (9). He identifies three fundamental types of skateboarding spaces:

**Found space**—architectural elements within urban environments that were not originally intended for skateboarding but are rendered skateable, often repurposed as *street spots*.
**Professionally purpose-built space**—designated areas specifically engineered for skateboarding activities, including skateparks, indoor skatepark, and skate plazas.
**Self-built space**—structures created by skaters through *DIY* (do-it-yourself) practices, typically informal and frequently unauthorized projects of varying magnitudes.

In addition to these fundamental spatial categories, further concepts are explored based on the creative technique of the “Morphological Box” (71), aiming to generate new solutions through the systematic combination of parameters (Table 1). In the matrix provided below, Peters' fundamental space types are combined. Additionally, the table examines which new forms of concepts could emerge if the city provides substantial support or legally authorizes the official use of areas for skateboarding beyond conventional skateparks.

### 4.2 Definitions of skateboarding space concepts

**Skatepark^7^**—a purpose-built, artificial space professionally constructed and optimized for skateboarding as well as related movement practices, typically comprising multiple obstacles. Skateparks are classified as sporting facilities, often situated in designated recreational areas of the city, and are structurally segregated with a clear material-spatial separation.

***Street spot* and *street legalized***—these are spaces not originally designed for skateboarding. Found spaces within the urban environment are transformed into skateboarding spaces through the creative reinterpretation and repurposing by skateboarders. The essential characteristic of any space suitable for skateboarding is a smooth surface. In this way, the entire urban environment becomes a potential skateboarding space (72).

*Street spot legalized* refers to skateboarding spaces explicitly authorized by the city for public use.

***DIY, DIY hybrid* and *DIY legalized*—**these are skate spots self-constructed by non-professionals, predominantly using *in situ* concrete. Operating on the edge of, or often beyond, legality, these *DIY* structures are often built incrementally on vacant lots such as industrial wastelands, transitional spaces under bridges, and even within inner-city areas. As urban interventions, these efforts modify existing *street spots* or creatively transform potential spaces, making them suitable for skateboarding (9). Occupying public or private spaces, altering them for personal use, and repurposing them for skateboarding is a defining characteristic of the *DIY* phenomenon.

*DIY hybrid* represents a collaborative effort between *DIY* practitioners and the city, where particularly dedicated skaters work in collaboration with municipal authorities to realize their *DIY* construction projects. These projects are typically overseen by professional skatepark builders and must adhere to specific legal regulations (see below).

*DIY legalized* refers either to a *DIY* project retroactively approved by the city after being initially constructed illegally, or to cases where the city provides a site for a *DIY* group prior to construction, allowing them to build under *DIY* principles within predefined conditions. The size and structural integration of *DIY hybrid* and *DIY legalized* spaces into the urban fabric often resemble skateparks.

***Shared spot****—*refers to multifunctional spaces. This concept is based on the intentional creation of multifunctional urban furniture and shared spaces. Skateboarders and other citizens “share” the use of selected “spots” within the city. *Shared spots* function as urban furniture, such as benches, railings, stairs, or sculptures. Typically, a *shared spot* is a materially modified, repurposed space within the public urban environment, partially designated for skateboarding. These spaces are designed for both skateboarding and other uses, without rigid boundaries or singular purposes, allowing for shared, multifunctional interaction. For example, a skateable granite block may also be planned and utilized as a bench for pedestrians. A defining feature of *shared spots* is the dissolution of clear spatial-material boundaries, fostering a more inclusive and adaptable use of public space.

The selection of the following *models of good practice* focuses on *DIY legal*, *DIY illegal*, *DIY hybrid*, *shared spots*, and legalized *street spots*, which are presented here as innovative, new, and creative skateboarding spaces.

### 4.3 Case studies: *DIY legal, DIY illegal*, and *DIY hybrid*

#### *Burnside,* Portland, USA

*DIY* projects no longer seem to contradict the official creation of spaces for skateboarding by municipal authorities (64). The *DIY* practice that has been established in parts of the scene for decades, which is characterized above all by a self-determined, mostly illegal appropriation of space and skateboarding artifacts that are successively produced on one's own initiative on derelict areas, under bridges, in transit spaces and similar non-places in the city (15), increasingly appears in a new light for urban policy and a new willingness to accept these *gifts* of creative urban design from the skateboarding scene. On the one hand, in some places, official authorization (*DIY Legal*) is granted retroactively for structures that were initially built without permission, such as *Burnside* in Portland, Oregon, USA (3), one of the world's most prominent *DIY* projects in the skateboarding scene. On the other hand, city administrations can leverage the strong commitment of the skateboarding community to *DIY* practices by integrating *DIY* skateboarding spaces into urban policy as legitimate projects.

#### *Lentpark*, Cologne, Germany

The city can make areas available for *DIY* use (often subject to conditions) or get involved in the construction project and the process as a developer in the form of a hybrid *DIY* project.

As an example of the latter concept, a *DIY hybrid* park was created in Lentpark in Cologne as a supplement to the city's existing, extensive skatepark offering, in which a derelict roller hockey pitch was realized as a *DIY* project in close collaboration with the city under the professional guidance of a skatepark company and the strong commitment of local skateboarders (9, 36, 38).

### 4.4 Case studies: *shared spot* and *street spot* legalized

#### *Shared spot* Landhausplatz, Innsbruck, Austria

On Landhausplatz in Innsbruck, skaters and passers-by share an elaborately designed *shared spot* on around 9,000 square meters as a communal space in the city center, which was not initially planned as such, but developed its own momentum through the users and became a very lively square (73).

#### *Shared spot*, Vigo, Spain

In Vigo, in the south of the Atlantic coast of Spain, an unintentional but no less effective *shared spot* was also installed in the Plaza de la Estrella. In a relatively large square, structured by three rectangular green areas and featuring a smooth stone floor, a seating block approximately 20 meters long and 1 meter wide was placed in one of the two thoroughfares. This urban piece of furniture is also made of smooth stone and is thus integrated into the materiality of the surroundings. It has no metal edge protection or other material clues that would indicate a specific function such as a skatepark element.

#### *Born Skate Plaza*, Barcelona, Spain

In Barcelona, Spain, the Born Skate Plaza was built in the middle of the trendy Born neighbourhood. To this end, an elongated area was installed as a widening of a sidewalk without any overly obvious area demarcation with smooth stone slabs as flooring and knee-high granite blocks in the public space.

#### Place de La République, Paris, France

In the heart of Paris on the Place de La République, skateboarding is not only legal, but skateboarders have been allowed to permanently install skateboard elements as art objects in certain areas of the large public square, which also qualifies the square as a *shared spot.* The Place de La République is literally a “Republic Square”, in keeping with the cultural-genetic city type of the Central European city. As a democratic forum, it provides space for rallies, demonstrations, and other forms of civic participation, as well as a permanent spot for skateboarding in the center of Paris.

#### *Southbank Undercroft*, London, United Kingdom

In another global metropolis—London—the city has allocated urban space to skateboarders in the form of the Southbank Undercroft, despite the significant economic potential for further commercial development, such as retail shops. The mayor at the time, Boris Johnson, recognized the cultural potential of skateboarding and justified the decision in favor of the users by stating that skateboarding is part of London's culture and that this location attracts tourists from all over the world, who undoubtedly contribute to the vibrant life and help to “make London the great city that it is” (36). What is remarkable about this example is that instead of offering skaters an alternative purpose-built space to the Southbanks, the skate spot remains where it is.

#### *Legitimized street skateboarding*, Bordeaux, France

On the Atlantic coast of southern France, the mayor of Bordeaux is particularly keen to ensure that “young people play an active and equal role in the city” (74), (min. 15:05). This sensitization to the social participation of young people, taking their wishes and needs into account and recognizing them for their commitment has led to the integration of skateboarding as part of urban planning. A city like Bordeaux had previously spent money to prevent skateboarding by installing skate stoppers. Now they are pushing for the money to be spent on integrating and legitimizing skateboarding into the cityscape (ibid.). By using public space in this way, the city shows its attitude towards the needs of young people: “Skateboard spaces are increasingly a way in which a city can speak of its attitude to youth, creativity and public space” (64). The commitment of skateboarders and the collaborative attitude of the city have now led to a fruitful collaboration that has resulted in a wide variety of spaces for skateboarding in Bordeaux. In addition to a wide range of skateparks, skateboarding has been (re-)legitimized in large public spaces and established in the sense of *shared spots.* As part of a city-funded art project, creative skate artifacts were distributed throughout the city center along the Garonne and integrated into the public space. Through these diverse manifestations of spaces for skateboarding, the practice became firmly anchored in the cityscape.

#### *LOVE Malmö*, Malmö, Sweden

In Malmö, Sweden, in addition to many skateparks, legal *street spots* (e. g. Svampen)*, shared spots* and designated places for *DIY* practices (e. g. TBS “Train Banks Spot”) are also part of an overall municipal concept (75, 76). Part of the concept is the medium of art in the form of a skateable sculpture. The sculpture arrangement was designed as a work of art by US professional skateboarder Alexis Sablone and installed in a public square as a *shared spot* (77) and authorized for use by various action sports.

With *LOVE Malmö*, the city located by the Öresund Bridge recently inaugurated another outstanding project. As an extension of the city's existing inventory of *shared spots*, a section of the world-renowned skateboarding cultural heritage site, the John F. Kennedy Plaza—commonly known as *Love Park* (see below)—was meticulously reconstructed using original polished granite slabs and blocks. At the Johannes Church in Malmö's city center, this *shared spot* was introduced under the title: “*FROM LOVE PARK TO LOVE MALMÖ.”* The spot comprises a linear, stage-like platform. On one end, it is connected to the ground with a narrow slope or wheelchair ramp as a driveway, while on the other, three steps form the end to the existing ground-level area. The platform is equipped with several seating blocks placed one behind the other, which are structurally arranged as tree planters and blend harmoniously into the cityscape. The skateboard cultural-historical context imbues this *spot* with a narrative that makes it particularly special for the local skateboarding scene and skate tourists, creating unique points of connection for the community to and identify with^8^.

#### *U.N. Plaza*, San Francisco, USA

With the recently created *shared spot* at *U.N. Plaza* in downtown San Francisco, California, USA, the integration of skateboarding spaces into existing urban architecture has reached a new dimension. The local skateboarding scene was involved in the planning with the participation of local users, a cooperation with the Bay Area-based *Thrasher Magazine*—world's biggest skateboard magazine—and the commissioning of a relevant skatepark construction company. On an area of 1,200 square meters, an arrangement of skateboarding elements was installed in the public space, paying homage to iconic *street spots* from the surrounding area and skateboard obstacles, some of which can also be found in skateparks. Following the existing architectural structure and materiality, the skateboarding elements were embedded in the aesthetics of the surroundings.

#### *Skate Melbourne*, Australia

Finally, the ambitious concept of the Australian metropolis of Melbourne should be mentioned. With the “Skate Melbourne” project, the city has undertaken nothing less than to structurally integrate a holistic municipal skateboarding space offering into its urban planning and thus make the entire city skateable (79).

For further examples of *shared spot* approaches and their characteristic design as well as the requirements for the surrounding area, please refer to the studies by (13, 72, 80) and (81).

As shown, these innovative spatial concepts offer a much more diverse spectrum of movement spaces that utilize additional urban potential. Between fiction and reality, approaches to solutions become apparent here that, in the context of a subcultural/free (*street spots*) and sportified/tamed (skateparks) form of skateboarding (82), also correspond to the socio-cultural logic of the skateboard scene, whereby they can address the wishes and needs of large parts of the scene (12, 44, 45). The examples also bear witness to a change in the public perception of skateboarding. Whereas in the 1990s, skateboarding on the streets was “virtually a symbolic vehicle of urban decay” (39), a “symbolic pollution” (83) and “[…] threat of disorder” (ibid.), the most recent concepts selected here show an unprecedented valorization of the practice in that city, which in the broadest sense is aimed at the creativity and distinctiveness mentioned above. The *U.N. Skate Plaza* project in San Francisco is remarkable, as never before has a *shared spot* skateboarding space concept been so explicitly linked to positive revitalization—“to transform the center into a safe, clean, and vibrant public space” (84), (min. 1:36)—and the intended safety through social control, which is expected to go hand in hand with revitalization, primarily through skateboard use. However, from a critical standpoint, the case illustrates how the skateboarding scene is being instrumentalized as part of a neoliberal urban policy strategy, particularly in light of unsuccessful attempts to address the “[…] drug crisis zone” (85) at *U.N. Plaza*. The city's earlier stance, marked by a complete ban on skateboarding at skate cultural significant spots, such as *Embarcadero*, *Pier 7*, and the *Library*, reflects a historically repressive attitude. This shift suggests that the city council has prioritized the mitigation of perceived negative externalities, choosing what they perceive as the lesser of two evils, rather than proactively considering the social and cultural benefits of incorporating skateboarding into public spaces. This phenomenon can also be interpreted as a Trojan Horse: while the city's apparent acceptance of skateboarding culture may appear genuine, it likely conceals a more covert agenda, serving a regulatory purpose aimed at exercising control. Without intending to pass judgement on the approach itself, the question arises as to how sustainable such projects can be within the context of the relationship between the city and the local skateboarding community.

It seems as if the connotation of skateboarding is gradually being reframed by the cities (15). The many examples mentioned also illustrate the increased willingness of the skateboard scene to collaborate, something that would have been barely conceivable in the early 1990s during the emergent phase of street skateboarding.

## 5 Results

### 5.1 Requirements for innovative skateboarding space approaches

The integration of official spaces for skateboarding in the city must be seen as a prerequisite in many respects. After all, cultural, socio-political and economic aspects play an important role.

If we assume that the creation of spaces for skateboarding requires a *bottom-up* initiative, there are several factors that must be considered in the dialogue process with city officials. These include, but are not limited to, the extent to which local skateboarders are able to articulate the need for skateboarding spaces beyond skateparks, and their ability to communicate the benefits of such spaces in a way that is both persuasive and trustworthy. The decisive question is then whether the city is willing to utilize the creative potential of the actors and is prepared to implement this citizens' request into the structures of existing urban planning in a socially acceptable and creative way. While the planning of skateparks often involves filling existing urban planning structures with new content—such as skateparks as recreational facilities—alternative skateboarding space designs, such as DIY projects in unique spaces, require a more profound influence on the existing procedures and conventions of urban planning, especially when implementing skateboarding space concepts like shared spots. Prominent examples of public negotiations and failed attempts to avert the threat of skateboard bans in city squares and officially legalize skateboarding there illustrate that this is precisely where the structural difficulty lies. In Philadelphia (USA), the skateboard ban on the skateboard-iconic *John F. Kennedy Plaza* (*Love Park*) (58) is compensated for with the *Franklin's Paine Skatepark* (86); in Chicago (USA), the city's *Leisure City approach* refers exclusively to purpose-built spaces, which, as already mentioned, reinforces the criminalization of *street spots* (3); and in Cologne, the *Kap 686 Streetplaza* is being created as an alternative to the *Domplatte*. The fundamental criticism of these approaches can be illustrated by the example of Peters and Schweer, who criticize the city of Cologne's policy of banning skateboarding on the *Domplatte* for good reason, with the former stating that it depends on the “prevailing guiding principles of urban development whether skateboarding practice is criminalized and persecuted as deviant behavior or promoted as an agent of culturalization of a *creative city*” (9) and the latter dubbing the actions of Cologne's skateboarding scene a “neo-liberal self-design” (36). This is because the tried and tested urban policy instrument of compensating for skateboarding spaces with alternative skateparks falls short because segregation from public space neglects the needs and wishes of many skateboarders (4, 9, 81) and the activation of urban creative potential in equal measure.

Moreover, the cases of Philadelphia and Cologne demonstrate how attempts to legalize or further tolerate *street spots* often face substantial challenges, leading to asymmetrical substitution practices. For instance, inclusive *street spots* are replaced with segregated skatepark infrastructures. This phenomenon exemplifies a “neoliberal bargain” marked by a coercive “take-it-or-leave-it” rationale.

The case of London's Southbank illustrates a completely different approach, thanks to the commitment of the *Long Live Southbanks* initiative. Instead of giving in to the pressure of economization and building a skatepark somewhere in the urban space to compensate for the historic Southbank skate space, former mayor Boris Johnson decided that the space should remain where it is (36). This example is groundbreaking in that it shows that it is also possible to overcome the difficulties of urban planning integration on a larger scale. According to the London *Long Live Southbank* initiative (87), securing space for users can be read as a victory of “culture over commerce and community over capital” (36).

It is easy to understand that the more the places designated as functional spaces for skateboarding are abandoned, the more permeable the contours of alternative spatial concepts become, and the more they move into the social spaces of public squares, the greater the potential for spatial conflict. Furthermore, the principle can be formulated that the greater the projected spatial conflicts, the greater the difficulty of officially enforcing *shared spot* use or legalization in socio-political terms, not to mention the legal requirements for bringing such a sporting opportunity into the circulation of public space. Finally, the development of creative spatial concepts and solutions necessitates a minimum level of “liberal openness,” tolerance, and social acceptance from citizens (think of the numerous Scandinavian *models of good practice* in urban planning). However, these conditions can vary significantly between countries, cities, and even districts within federal systems. These cultural differences should not be underestimated, as they can represent a decisive opportunity factor. For example, when a relatively large proportion of people in less significant everyday situations tend to pedantically insist on their formal and informal rights as a matter of principle, even if this is at the expense of harmonious coexistence. This behavioural structure can take on a greater dimension if people do not shy away from legal action and the organization of citizens' initiatives aimed at preventing spaces for skateboarding. It is obvious that such a “socio-cultural climate” can lead to city administrations reacting in advance with less courageous and opportunity-oriented action, as the potential “procedural error” and legal consequences hover over them like a sword of Damocles. Without being able to pinpoint this phenomenon scientifically, tolerance and an interest in harmonious conditions do not appear to be equally pronounced in all countries and cities, although they can have a significant effect on the realization of innovative skateboarding space implementations. Different urban cultures either expand or constrain opportunities for action at a superordinate level and must therefore be taken into account. It is important to mention that “a critical factor being integrated into the city life is that skateboarders realize they must be good partners to the city” (88). In other words, regardless of general socio-political conditions and the size of the city, the successful realization of such alternative skateboarding space projects is often the result of civic engagement and a sometimes long-term continuous mutual relationship between committed skateboarders and city representatives (13, 75, 81).

Although the city would theoretically have many reasons—even beyond those mentioned here^9^—to implement alternative space concepts to skateparks in the city, the emergence of many *shared spots* and legitimized, found space examples indicates that an initiative *from below* is also needed here.

### 5.2 Found street spots vs. shared spots

It might even be possible to intentionally create skate spots that are legitimate within the scene. This means that they are perceived as found spaces. This is because, from the scene's perspective, sustainable symbolism that meets its standards of authenticity is ultimately linked to the credibility of integration into the public space and the concrete implementation in the urban structure and aesthetics. It concerns street furniture that is explicitly not primarily derived from the sporting and functional needs of skateboarding, but results from other contents and functions that are part of the contingency of urban architecture in their appearance (seating, borders, boundaries, edging, connecting differences in height with paved surfaces as slopes, works of art such as sculptures, installations, etc.) [see also (13)].

### 5.3 Skateboarding space concept positioning model

The final section conceptualizes the different skateboarding space approaches in a positioning model based on the preceding considerations and explanations.

**Figure 1:**
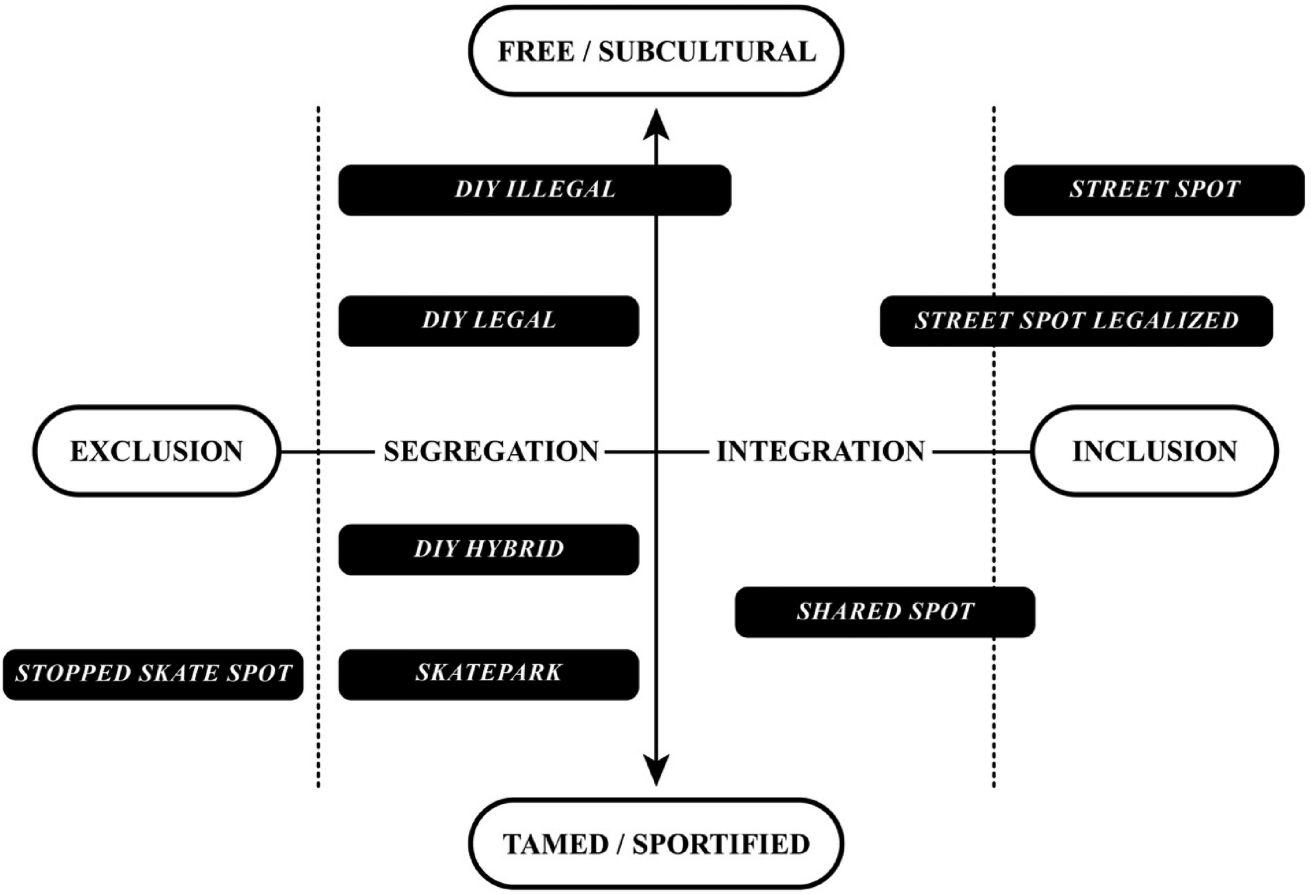
Skateboarding space concept positioning model. Source: own presentation.

In the positioning model (see Figure 1), the spaces mentioned are positioned between the aspects of exclusion and inclusion as well as the degree of freedom of the respective skateboarding space concept. The idea of inclusion, which is otherwise primarily used in social contexts, especially social participation, and the associated relational concepts such as exclusion, segregation, and integration, is transferred here to spatial-material spaces. The approaches discussed represent different spatial-structural dispositives for skateboarding, in which relation they are part of the urban space, from prohibitions (exclusion) to complete fusion with the city as found *street spots* (inclusion). The degree of freedom relates to spatial concepts that are associated with a higher level of “tolerance for ambiguity” (89). Skaters often practice a more creative and interpretative approach to the utilization of public space, whereas city officials typically adhere to structurally predefined and functionally narrower conceptions of spatial use (ibid.).

The model is structured by a horizontal and a vertical axis that intersect in the center. The horizontal axis indicates the character of the spatial concept and is divided into four sectors, from “Exclusion” to “Segregation”, “Integration” and “Inclusion”. On the vertical axis, the degree of freedom of the respective spatial concepts is shown in the tension between the poles of “Free/Subcultural” and “Sportified/Tamed”*. “*Free/Subcultural” spatial concepts are those that tend to serve an unstructured and unregulated use. Whereas “Tamed/Sportified” skateboarding spaces tend to have contoured and structured characteristics.

The spatial concepts of the positioning model can be distinguished based on the following two primary criteria.

#### 5.3.1 Land use plan and area designation

The presented best practice models demonstrate that skateboarding spaces can be incorporated into various zones within a city's land use plan. These spaces may be located in traditional recreational and sports areas or in more unconventional locations, such as brownfield sites, under bridges, or in gaps between buildings. Additionally, the architecture of public urban spaces can be adapted for skateboarding purposes.

In conclusion, the more a skateboarding space blends into the typical urban environment, the more it tends toward “Segregation” on the horizontal axis and “Teamed/Sportified” on the vertical axis. In contrast, spaces with a non-conformist urban location move higher on the vertical axis toward “Free/Subcultural” and toward “Integration” and “Inclusion” on the horizontal axis.

#### 5.3.2 Spatial contouring and structural integration into the city

The exploration of various skateboarding spaces as case studies revealed that the spatial contouring of skateboarding spaces within a given area can take diverse forms. This refers to the nature of spatial-material integration—whether the skateboarding space is enclosed or demarcated, for instance, fenced off or enclosed through other design elements; partially permeable, perhaps separated from surrounding pathways by color, tactile markers, or spatial divisions; or fully open and integrated without any physical barriers. This means the more a skateboarding space is characterized by being structurally enclosed, isolated, fenced in, or sealed off, the more it tends horizontally toward “Segregation” and vertically downward toward “Teamed/Sportified”. Conversely, the more open, spatially integrated into urban life, and structurally permeable the skateboarding space is, the more it aligns horizontally with “Integration” and “Inclusion”, and vertically with “Free/Subcultural”.

### 5.4 Positioning of the skateboarding space concepts

The individual spatial concepts are ideally located within the positioning model: In the “Segregation” sector, *DIY* spaces and the classic skatepark are primarily positioned as spatially clearly defined and delimited facilities. *DIY Illegal* and *DIY Legal* tend towards the pole of “Free/Subcultural” in the upper area, whereas *DIY hybrid* and Skatepark move towards “Tamed/Sportified” in the lower area. The “Integration” section contains only the concept of s*hared spots*, which primarily comprises projects that are integrated into the public space—if at all—only with very low-threshold, barely visible spatial-material boundaries. Spatial concepts with the character of “Inclusion” are officially legalized or at least tolerated *street spots* that are freely and openly integrated into the urban space without explicitly intended spatial separation.

It is important to note that the skateboarding space concepts are not rigid units, but sometimes occur in mixed forms, so that the transitions from one concept to another can be fluid or overlapping, as indicated in the model^10^.

The skateboarding space concept positioning model illustrates the extended options of action for cities and municipalities beyond skateparks to create spaces for skateboarding and how they are positioned between exclusion and inclusion as well as how they can be located in the field of tension between the poles of subculture and sportification.

## 6 Discussion of the possibilities and challenges of new skateboarding space concepts

The many examples of skateboarding spaces in the context of the *creative city* should not obscure the fact that they are currently still the exceptions (62) and that skateparks are—as before—the rule of urban policy when it comes to creating spaces for skateboarding. The utopia that, in the future, spaces for skateboarding will be woven entirely into the public space of cities as explicit or implicit *shared spots* and that in this way the once segregated purpose-built spaces in the city will largely become fluid is highly unlikely. Given the diverse needs of skateboarders and urban governance, the future does not appear to herald a post-skatepark era but rather seems poised to be defined by an increasing diversification of skateboarding spaces. Based on the findings of this study, there is evidence to suggest that a varied provision of skateboard spaces as part of a city-wide planning strategy will adhere to a both/and logic, rather than an either/or approach. As skatepark concepts become more diversified, *DIY spots* can no longer be viewed merely as stopgap measures in many locations, but rather as ends in themselves.^11^ The increased spread and presence in cities that offer a comprehensive and diverse range of spaces for skateboarding indicate that *DIY* projects should be seen as a diverse addition and tend to be seen as a complementary part of a municipal skate space offer. The ongoing evolution of spaces for skateboarding appears to be leading to heterogeneity and diversity (90).

Found spaces for skateboarding appear to hold a central role within the skateboard community; however, the advantages of skateparks, particularly their social dimensions (see above), should also be taken into account in municipal planning. A not insignificant question here is whether the skateboard scene can reach a consensus on a common objective, specifically which skate space needs they aim to address, and what capacities exist for medium- to long-term political engagement at the municipal level to support this cause. What kinds of skateboarding spaces are they interested in creating? Are they focused on legitimized *street spots*, *DIY spots*, *shared spaces*, or do their personal preferences lean toward traditional skateparks?.

## 7 Conclusion

In conclusion, the study demonstrated how new concepts of skateboarding spaces were scientifically defined and conceptualized, addressing the first research question. The “Skateboarding space matrix” facilitated the identification and theoretical formulation of these spatial concepts, which were subsequently advanced into the “Skateboarding space positioning model,” providing a framework for practical implementation. From an urban sociological perspective, these new spaces particularly relate to the concept of the *creative city*. It should now be evident that skateboarding can contribute positively to urban environments in various ways. However, the imposition of skateboard bans and the uncritical segregation of skateboarding into purpose-built skateparks as a singular course of action risks exacerbating the criminalization of natural *skate spots* and informal sporting opportunities. As demonstrated, such measures can obscure the urban creative potential found in *DIY* projects, *shared spaces*, and legalized *street spots*. While acknowledging the fundamental justification and numerous positive aspects of skateparks, these new concepts often provide more integrative, inclusive, and collaborative approaches that align more closely with the (sub)cultural practices of skateboarding.

With respect to the enabling and limiting factors in response to the second research question, a key success factor identified was the relationship between an overall more established local skateboarding community and city representatives. Public skateboarding spaces are invariably the result of a dialogue, or more accurately, a collaboration between skateboarding initiatives and local authorities. The quality of this relationship is socially evident and materially manifested in the spatial concepts that are concretely implemented. The stronger the relationship, the more concepts can be found that fall under the categories of “Integration” and “Inclusion” as presented in the positioning model. It should be noted, however, that the generalizability of the practical implementation of the concept for creating skateboarding spaces is limited due to varying city politics and the capacities of local skateboarding space initiatives.

Building on the insights presented in this article, further research projects could employ empirical methods to investigate the emergence of innovative space concepts such as *shared spots* or legalized *street spots*. In the context of participatory city planning, it would be beneficial to examine the prerequisites, possibilities and difficulties inherent in the dialogue between the skateboarding scene and the city. How can pathways structurally be established that opens a continued relationship that might lead to collaboration in city planning (91). Furthermore, analyses of the social aspects of use—particularly in relation to marginalized user groups—as well as of the user groups themselves, could represent worthwhile endeavors with the potential to advance the current state of research. Finally, another area of scientific inquiry could focus on the environmental impacts of spatial structures designed for skateboarding. A particularly promising direction would be to explore how existing impervious surfaces in cities can be repurposed for *street* and *shared spots*, transforming them into multifunctional areas. These synergies could promote the development of more ecologically sustainable and environmentally friendly environments, while also reducing the ecological footprint of purpose-built skateboarding infrastructure.

The unequivocal recommendation of this study is a concept of diverse spaces for skateboarding as a comprehensive municipal offering. The findings of this research provide skateboarding space initiatives and city officials with an expanded range of options, extending beyond traditional skateparks, to identify the most suitable spaces for skateboarding within their city.

## Data Availability

The original contributions presented in the study are included in the article/Supplementary Material, further inquiries can be directed to the corresponding author.

## Footnotes

^1^Skate spots include all physical spaces that offer the opportunity to skateboard.

^2^To prevent anecdotal information, I focus on aspects and events directly related to skateboarding spaces.

^3^Especially for marginalized user groups who often seek safe spaces, temporary exclusive spaces can be created through special events or other forms of skatepark programming and regulations.

^4^It should be noted that the presentation of this theoretical concept of the *creative city* has a dualistic bias. In practice this concept is less clearly implemented, can be more nuanced, and is sometimes working under different labels.

^5^The protagonists of the Cologne skateboard scene in the study by (9), who organized themselves as officials in the association during the conflict over the skateboard ban on the *Domplatte*, are also over 30 years old.

^6^Skateboarding spaces with this quality include the *Peitruss skatepark* in Luxembourg, the MCBA *street spot* in Barcelona, the *shared spot* on Landhausplatz in Innsbruck, and the *Burnside DIY* park in Portland, Oregon, USA, among others.

^7^This refers exclusively to public skateparks.

^8^Refer to (78) and (62) for further information on the project.

^9^Discovery of previously overlooked places (…) inner cities as places of sensual play (…) sustainable mobility in the city (…) lack of movement and urbanization (…) culture of togetherness and encounters (…) skateboarding as a space of opportunity for political education" (15).

^10^Such as "integrated skateparks" (13).

^11^It should be noted that *DIY* projects require a special knowledge and commitment from the respective scene, which is not equally present in all cities.
